# Supplementary material for: Effect of the Size and Shape of Dendronized Iron Oxide Nanoparticles Bearing a Targeting Ligand on MRI, Magnetic Hyperthermia, and Photothermia Properties—From Suspension to In Vitro Studies
Source: Pharmaceutics. 2023 Mar 30;15(4):1104. doi: 10.3390/pharmaceutics15041104 (PMC10143744; doi:10.3390/pharmaceutics15041104)
Supplement: Supplementary file 1 [file pharmaceutics-15-01104-s001.zip › pharmaceutics-2224798-supplementary.pdf]

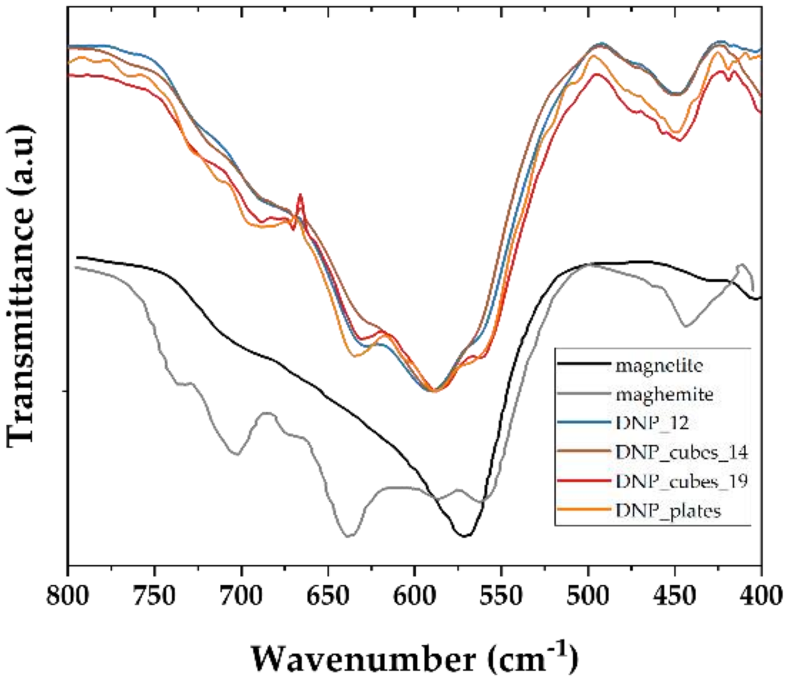

Figure S1. FT IR spectra of DNP_12, DNP_cubes_14, DNP_cubes_19 and DNP plates in the region 800 – 400 cm^{-1} corresponding the Fe-O band compared to spectra of magnetite and maghemite.

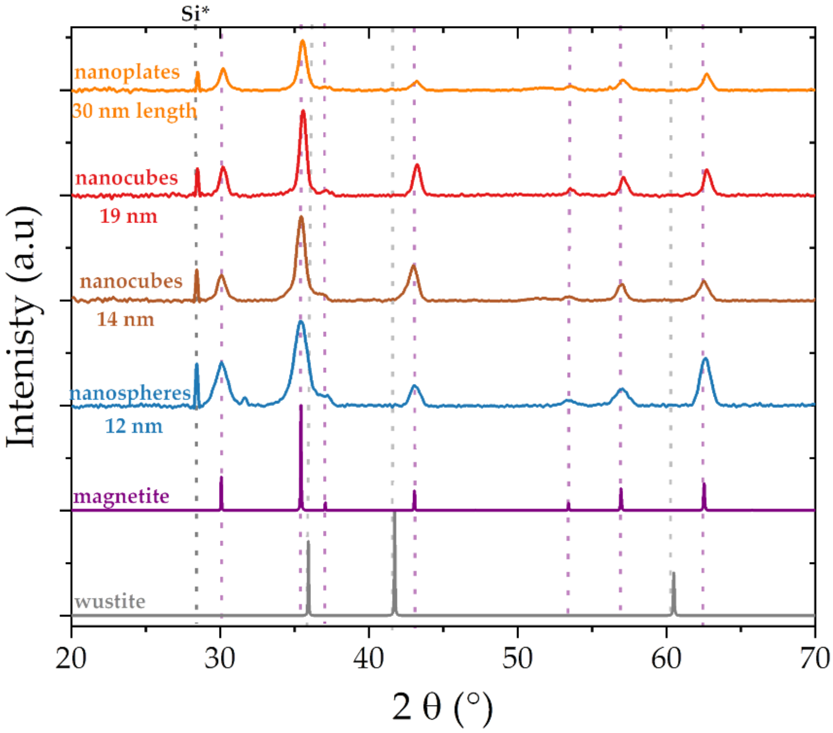

Figure S2. X-ray diffractograms of DNP_12, DNP_cubes_14, DNP_cubes_19 and DNP plates compared to theoretical diffractograms of magnetite and wustite.

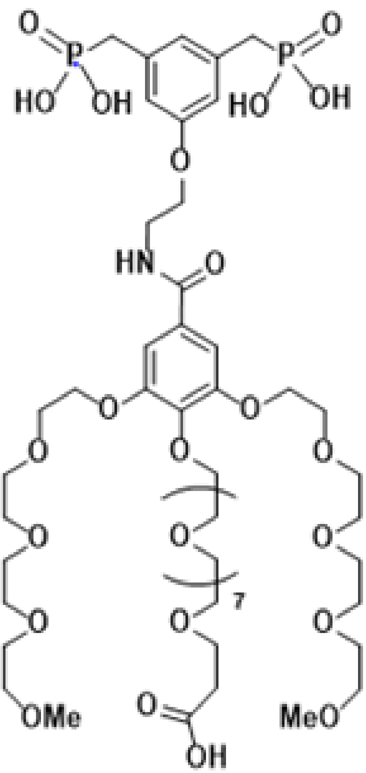

Figure S3. Chemical structure of the dendron molecule D1-2P used to obtain stable colloidal suspensions of NPs in water.

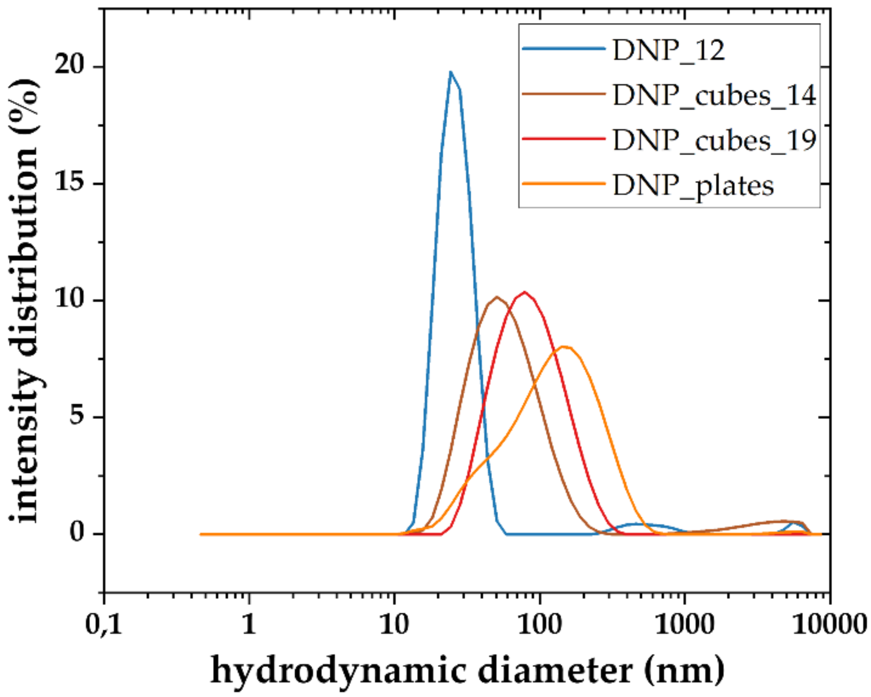

Figure S4. Hydrodynamic size distribution in intensity mode measured by DLS for all DNPs.

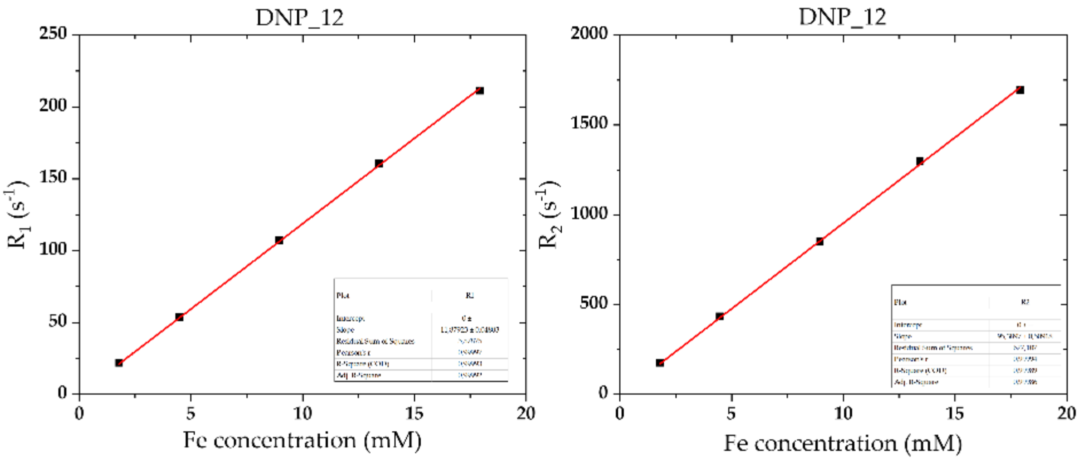

Figure S5. Relaxation rates R_1 (left) and R_2 (right) in the function of iron concentration for the DNP_12 suspension. A linear fit was made to assess the transversal and longitudinal relaxivities (respectively r_1 and r_2). The same procedure was applied for all the DNPs.

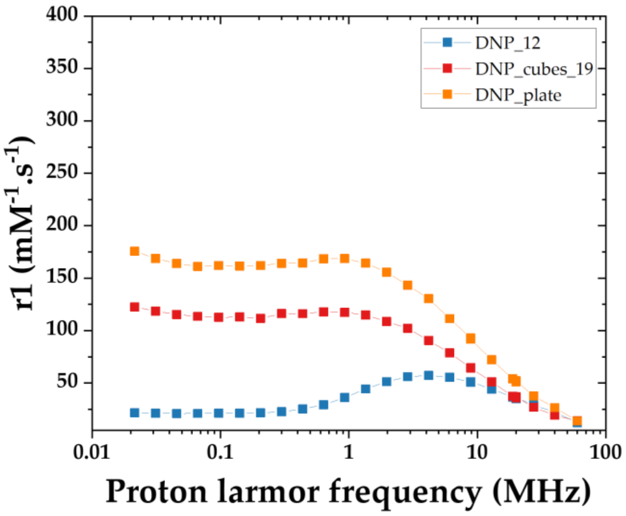

Figure S6. NMRD profiles of different DNPs.

Table S1. Extracted M_s values and NMRD radii after fitting of NMRD curves.

Batch	M_s (from SQUID data) (emu/g)	M_s (from NMRD) (emu/g)	NMRD radius (nm)
DNP_12	56	48	7.2
DNP_cubes_19	85	46.3	9.3
DNP_plates	57	53.3	10.1
